# Lidar reveals distinct insect daily activity and diversity between habitats

**DOI:** 10.1038/s41598-025-27432-9

**Published:** 2025-11-21

**Authors:** Assoumou S. Doria Yamoa, Benoit K. Kouakou, Adolphe Y. Gbogbo, Anna Runemark, Roel van Klink, Jeremie T. Zoueu, Mikkel Brydegaard

**Affiliations:** 1 Instrumentation, Imaging and Spectroscopy Laboratory, Félix Houphouët-Boigny Polytechnic Institute, Yamoussoukro, Côte d’Ivoire; 2 Department of Physics, University of San Pedro, San Pedro, Côte d’Ivoire; 3 Department of Biology, Lund University, Sölvegatan 35, 22362 Lund, Sweden; 4 German Centre for Integrative Biodiversity Research (iDiv) Halle-Jena-Leipzig, Leipzig, Germany; 5 Department of Computer Science, Martin-Luther-University Halle-Wittenberg, Halle (Saale), Germany; 6 Department of Physics, Lund University, Söllvegatan 14C, 22362 Lund, Sweden; 7 Norsk Elektro Optikk A/S, Østensjøveien 34, 0667 Oslo, Norway; 8 FaunaPhotonics, Oceanvej 1, 2150 Copenhagen, Denmark

**Keywords:** Biochemistry, Computational biology and bioinformatics, Ecology, Ecology, Environmental social sciences, Optics and photonics

## Abstract

Entomological lidar can provide rapid, non-intrusive assessment of insect abundance, diversity and composition, and has the potential to shed light on species specific preferences for habitats or environmental conditions. However, few studies have to date demonstrated comparisons of insect diversity and abundance across sites using photonic methods. Here, we present a first comparative entomological lidar study spanning different habitat types, investigating if the method can uncover consistent differences in insect diversity and composition. Using a simple, robust and parameter free estimation of entomological signal diversity, we recover the largest total number of insect observations (1,716,362), the highest daily number of observations (346,581) and the highest number of clusters distinguishable from noise (353) reported to date. We demonstrate consistent differences between four sites in terms of abundance, diversity, composition, range distributions and daily activity patterns. We critically discuss possible biases, constraints and future challenges for physicists and ecologists employing entomological lidar for diversity estimation.

**Supplementary Information:**

The online version contains supplementary material available at 10.1038/s41598-025-27432-9.

## Introduction

Recent research has highlighted a strong global decline in insect abundance and diversity^1–3^. The implications are wide-ranging and include reduced functioning and resilience of our planet’s ecosystems and our own crop production^4^. The decline is attributed to a multitude of causes, including habitat destruction, pollution, invasive species and climate change^5^ and is most severe for terrestrial insects in industrialized regions^3,6^. However, although the tropics harbor the highest insect species diversity, studies of tropical insect diversity and population trends are starkly underrepresented. This lag in documenting diversity and its decline for tropical habitats, resulting from limited research funding in many countries in the tropics, may lead to failure to identify threats with delayed intervention and possibly irreversible ecosystem damage as consequences^7^. Biodiversity surveys^8,9^ based on trapping and manual taxonomic classification are costly and labor intensive^10^, highlighting the need for automation^11^ or genetic^12^ approaches to detect threats to insect diversity. Approaches to online in situ monitoring of insect diversity^13^ include e.g. distributed acoustic devices^14–16^ and machine vision setups^17–19^. However, neither of these approaches are broadly applicable across insect orders and they are currently not able to capture a representative composition for insect species richness assessment. Whereas the total biomass can be estimated by weighing catches^1^, and species richness can be evaluated by e-DNA or barcoding^20^, the species abundance distribution (SAD) is exceedingly challenging to determine for insects. Current modern approaches include individual sequencing^21^ or robotic sorting and microscopy^11^.

Photonic entomological sensors^22–25^ detect free flying insect in situ throughout the day without any attractant or particular trap design. The aerial insect density can be determined quantitatively when the details of the probe volume is known^24,25^ Such systems typically pick up insect oscillations, which include a wingbeat frequency (WBF) and harmonic overtones^26,27^. Species and sexes with distinct WBF can thus be differentiated^28–30^. Even species with similar WBFs can be distinguished by the overtone characteristics which relate to the wing dynamics^31^, surface roughness^32^ and the wing membrane thickness^26,27,33,34^ in relation to the wavelength of the sensor. Differences in overtone content, rather than just frequency, is analogous to the distinction in timbre between a flute and a trumpet playing the same note.

Among photonic sensing, entomological lidar is particularly efficient in counting great numbers of insects passing through a laser beam (photon economy is optimal in lidar because the same light has high chance of intercepting an insect along a transect). However, it is generally challenging to associate the echoes with known taxa verified by other means. Nevertheless, numerous distinct signal types can be classified from field observations using unsupervised hierarchical cluster analysis (HCA)^35,36^. In these reports, light modulation spectra (including scattering from bodies, wings and their harmonics) from remote insects form the basis of a pairwise comparison, whereby the ensemble of observations can be clustered in groups of similar modulation spectra. Based on HCA, some of the clusters retrieved can be identified as male and female mosquitoes, although the majority of the clusters had lower WBF and remained unidentified^35,36^.

Depending on instrument complexity in terms of spectral- and polarization bands, similar species may not necessarily produce distinguishable oscillatory signals^28,29^. Furthermore, a single species and sex can be expected to produce distinct signals depending on the observation aspect^26,37–39^, temperature^40^ or payload^41,42^ (e.g. pollen, nectar, eggs or blood meals). Despite these reservations, a diverse ensemble of insects should also display a diverse ensemble of signals, whereas an ensemble with poor species richness should display only a few types of oscillatory signals. Initial work aimed at determining the number of unique clusters in entomological lidar data^43^ could discern 12 different signal types. A sensor based study^44^ demonstrated 70% correlation between photonically sensed insect clusters and insect family richness identified in co-located Malaise trap catches. This correlation is as high as that among adjacent Malaise traps^45^. To harness the full potential of entomological lidar, a key question is whether the number of discernible clusters are determined by the algorithms used, the instrument employed, or the habitat monitored, thus reflecting true biological differences. Revealing to which extent entomological lidar can provide insights into species abundance distributions, as well as the relationship between insect number and diversity are other major challenges. To address these questions, determining if there are consistent differences in insect abundance and diversity between different types of habitats, and how consistent and thus predictable abundance and richness values are from day to day is crucial.

In this study, we addressed whether or not we could determine consistent differences in insect abundance and diversity by deploying a near infrared entomological kHz lidar for four consecutive days in each of four distinct habitats. Daily insect counts ranged from tens- to hundreds of thousands of insects, and signals were clustered by pairwise Hierarchical Cluster Analysis (HCA). We investigated consistency between days and discrepancies in signal diversity across sites and explored cluster composition as well as the spatio-temporal distribution of the different clusters detected. We discuss possible biases and challenges in estimating biodiversity from lidar data in the light of our findings.

## Results

**Fig. 1:**
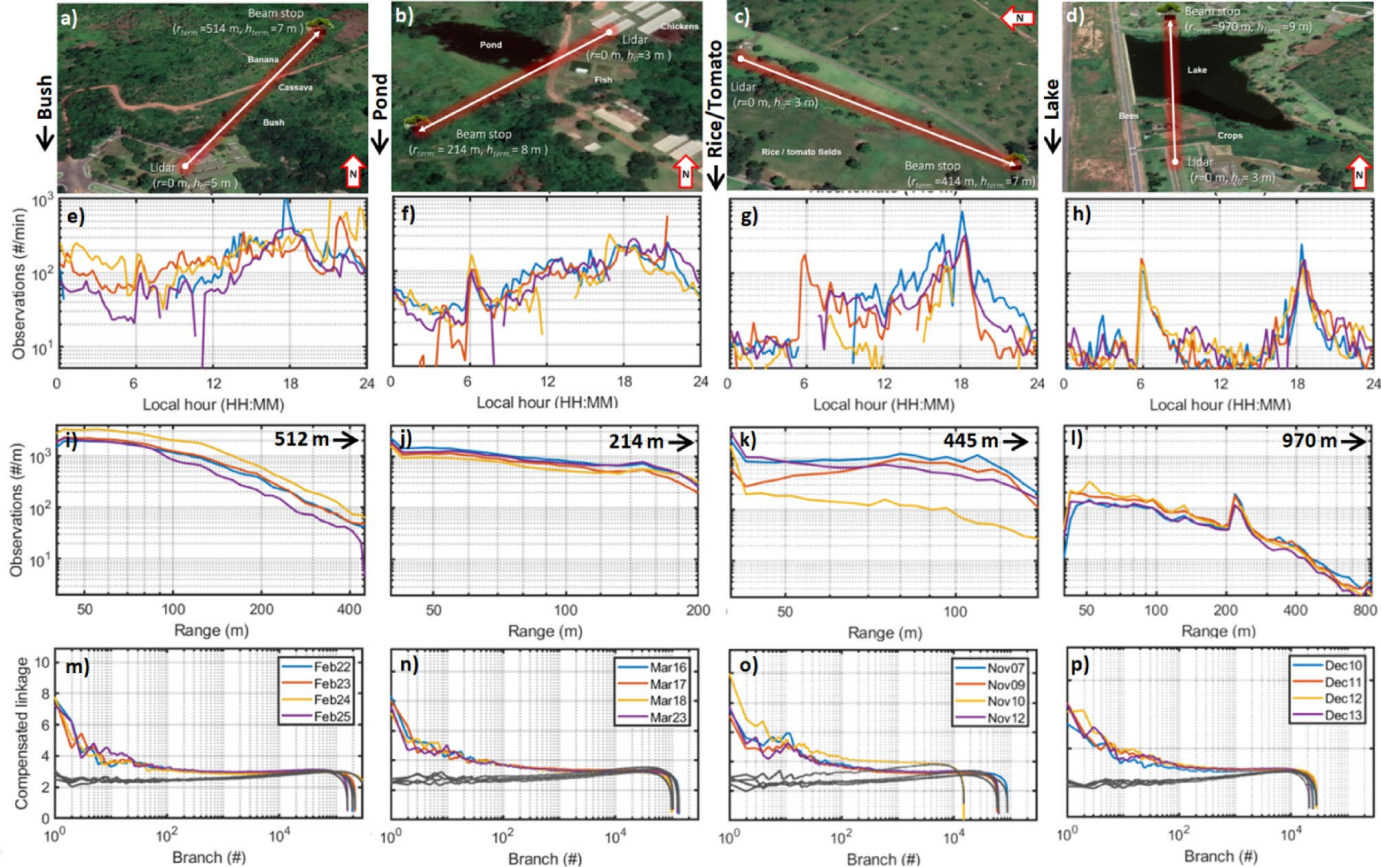
Focal study sites and lidar trajectories, insect abundance across time and space and inferred species richness (linkages). Top row: study sites, with lidar beam transects indicated by arrows. The beam length and height over ground is indicated in both ends. (**a**) Bush site, the beam passes > 7m high over scrubs and trees, including cassava, banana and mango. Termite mounds can also be found at this site. (**b**) Pond site, the beam is transmitted from a chicken farm and passes between a pond and fish dams. (**c**) Rice/tomato site, the beam passes close to small patches with rice and tomato cultures. (**d**) Lake site, the long beam passes between crop patches and beehives before passing over a lake. (**e**–**h**) The number of observations during the days for each focal site. The daily activity patterns are distinct among sites, but consistent among days. (**i**–**l**) The number of detected insect observations across the ranges of the beams. (**m**–**p**) Detrended signal dissimilarity linkage computed for each day and site independently. The larger values for the first branches on the left side indicate signal diversity, the drop on the right hand is a result of numerical precision of measurements and computations. The gray lines indicate equivalent linkages computed for real instrument noise recorded during the campaign, providing a negative control for signal diversity. Each column corresponds to the respective study site, and coloration denotes sampling date. (Map data: © Google, Maxar Technologies).

We deployed an entomological lidar^43^ to investigate insect diversity in Yammasoukro, Ivory Coast. We report a total of 1,716,362 individual insect observation recorded at four sites during 4 days for each site (Figs. 1a–d, S1-S). Insect signals (see definition in “Methods” section) were recorded, returning observation rates varying from 10 to 1000 observations per minute. The four sites show distinct daily activity patterns but predominantly consistency between the days (Fig. 1e–h). The bush habitat displays marginal variance during the day, while the ponds display minor morning rush hour and a broad peak around dusk, the rice and tomato patch show minimal nocturnal activity, whereas a strong crepuscular peak is seen at the lake site. The transects varied in length (Figs. S1–S7). While the general decay with range is associated with loss of signal, insects were detected at up to 800 m distance (Fig. 1i–l). Since the beams are elevated above the ground, the range profiles do not generally reflect preferences for topographic features. The last site, however, displays an activity peak at the shoreline of the lake.

Signals from each day and site are clustered independently by HCA (“Methods” section, Eq. 1). The compensated linkages (“Methods” section, Eq. 2), reflecting how dissimilar the modulation spectra among the groups are, decrease with the number of branches (Fig. 1m–p). The decay when branch numbers approach observation numbers imply that signals approach noise and numerical precision of instrument and computations. Instrument noise from real field conditions was used as a negative control, displayed in gray, and show no signal diversity (Fig. 1m–p).

**Fig. 2:**
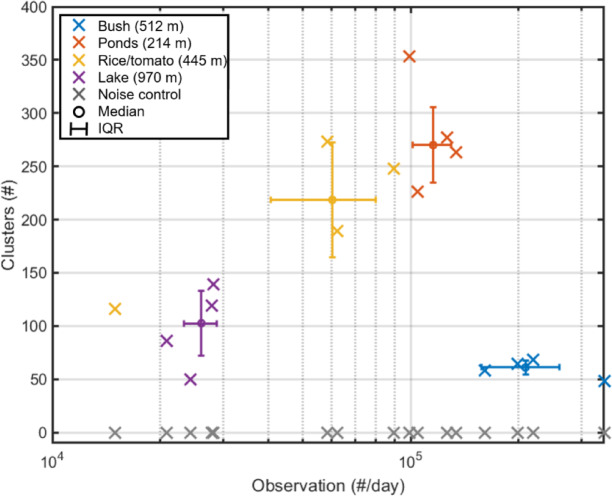
The relationship between number of observations and the estimated number of signal clusters. The number of retrieved clusters versus the number of insect observations. The points are colored by site, with gray representing a negative control based on signal noise. The median and IQR for each site are denoted by bars. Activity ranged from ~ 20,000 observations per day for the lake site to more than 300,000 observations per day for the bush site. The number of clusters that significantly different from noise ranged from 50 for the lake up 350 for the pond site.

In general, a clear cut-off in linkage, enabling to determine the exact number of clusters, cannot be expected for in situ data comprising thousands of species and different sexes and life stages. Moreover, WBF signals are affected by environmental conditions during the day. We identified the distinguishable number of clusters, *NoC*, for each day and site as the number of compensated linkages exceeding the median plus one IQR of the same compensated linkages (“Methods” section, Eq. 3). Applying this criterion, we did not detect any cluster for the negative control compensated noise linkages, *Z*_*ξcomp.(p)*_, for any of the sites or days. Thus, *NoC*, represents the number of clusters distinguishable from noise. The daily observation counts and daily numbers of clusters are displayed in Fig. 2.

In general, insect counts and *NoCs* were consistent within, but significantly different among, sites (Fig. 2). Interrupted measurements during one rainy day (Rice/tomato site, Nov. 10th) resulted in lower counts, and a windy day (Pond site, Mar. 17th) caused reduced transit times and reduced number of clusters, though. An asymptotic rarefaction curve, where species richness increases with number of observations is theoretically expected^46–48^. In our data, three of the sites displayed a positive correlation between number of observations and the number of clusters, consistent with other studies^49^ where too few individuals are sampled to exhaust the present pool of species. Therefore, extrapolation of rarefaction curves would be required to estimate asymptotic species richness^46–48^ also for our data, and as a high frequency of singleton is expected^45,50^ we likely do not detect many of the rare species. Interestingly, this relationship differed among the sites, with the bush site displaying the lowest *NoCs*, in spite of having highest number of observations likely reflecting that this site is dominated by a few species.

**Fig. 3:**
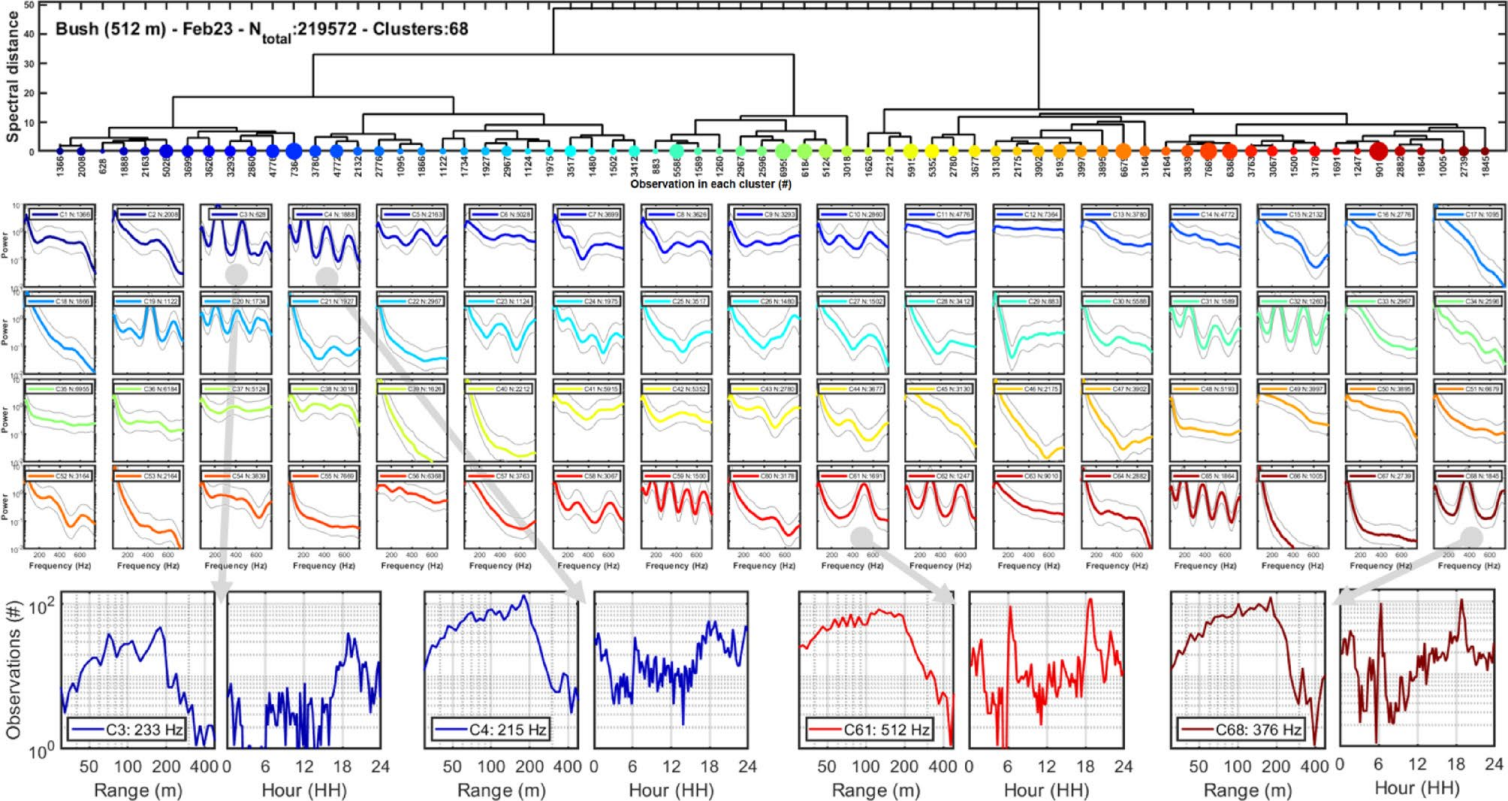
Example of hierarchical clustering based on a single day at a single site. Top; resulting dendrogram of signal relatedness. The branches are sorted by minimizing neighbor similarity. The sizes of the colored circles indicate how many observations are assigned to the cluster. Middle; the centroid modulation spectra for each cluster upon which clustering is based, where solid lines indicate the median spectra and grey lines indicate IQR. For several clusters a WBF can be deduced, multiple strong harmonics indicate glossy wings and clusters with same WBF but distinct strength of even and odd harmonics could result from same species observed from transverse or sagittal aspect. The most abundant clusters often show the least specific modulation spectrum which could result from them being umbrella clusters for multiple unresolved species. Bottom; the temporal and spatial frequency of occurrence for four selected clusters.

To illustrate the wealth of signal diversity we use a single day at the bush site as example (Fig. 3). The HCA clustering dendrogram (Fig. 3, top) displays the number of observations assigned to each cluster. The centroid modulation spectra are presented for the 68 detected clusters (Fig. 3, middle section). The clusters to which most observations are assigned also display the least specific power spectra dominated by low frequency content without prominent WBF or harmonics. This means that these are likely to be umbrella clusters grouping observations which cannot be resolved by the instrument due to short transit times or low WBFs. Time- and range distributions are extracted from each cluster (Fig. 3, bottom). The range distributions may indicate preferences for landscape features or detection ranges for the species in the cluster. The daily activity patterns differ among clusters, with the insects in some clusters being more active in the mornings, and others in the hotter afternoons. The insects in some clusters display sharp preferences for light level niches, with activity peaks during the short tropical dawns and dusks.

**Fig. 4:**
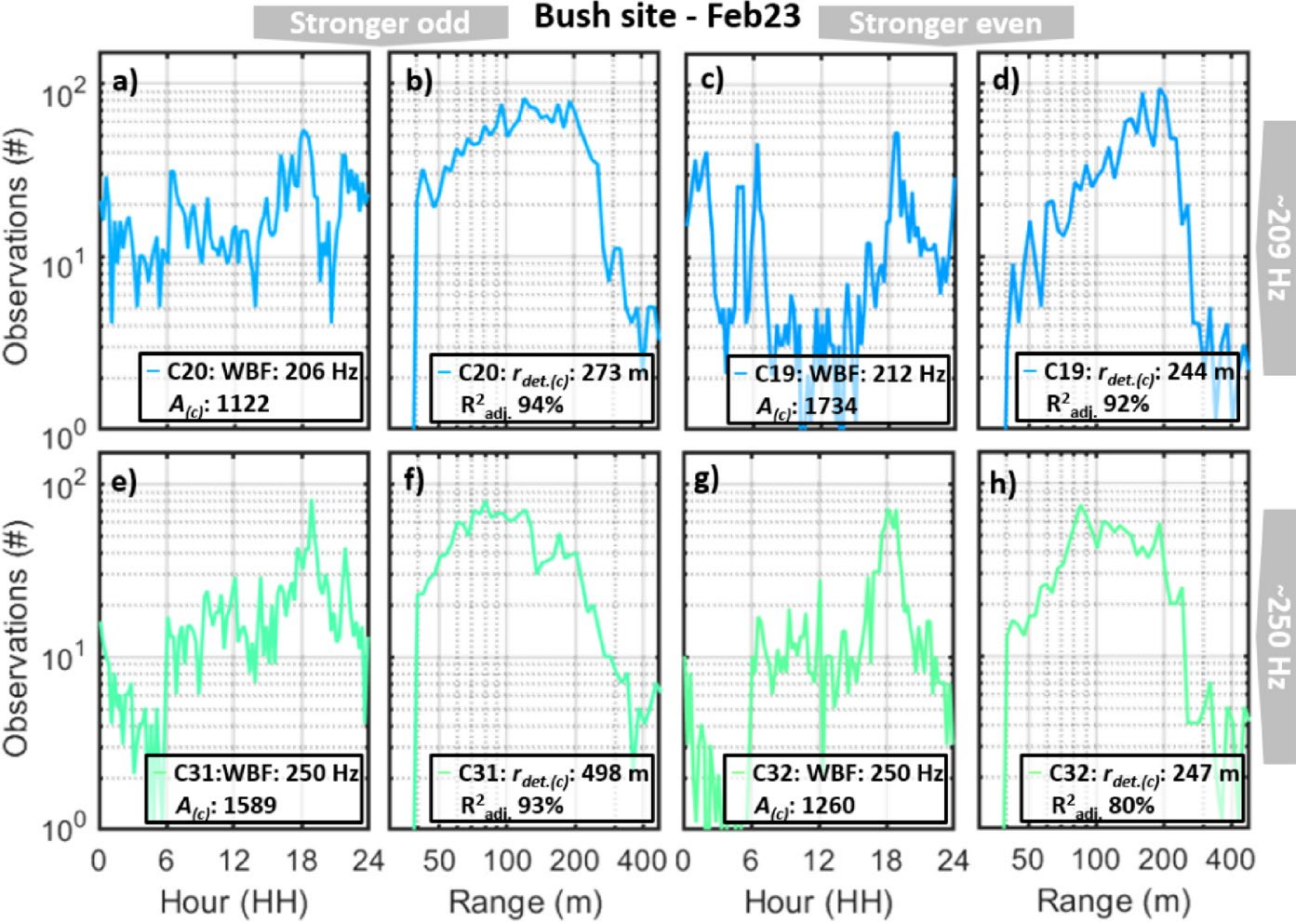
Time and range distributions of two pairs of clusters with similar WBFs but distinct harmonics. Clusters with strong odd harmonics (**a**–**b**; **e**–**f**) display higher counts and longer detection range than clusters with similar WBF but stronger even harmonics (**c**, **d**; **g**, **h**). This is counterintuitive since sagittal observations is both more likely and have larger cross sections but produce stronger even harmonics. Daily activity pattern also differs between these cluster pairs with similar WBFs but distinct harmonics.

Generally, this data shows that entomological lidar is a promising tool for diversity assessment and even potentially for estimating insect SADs. The number of observations in each cluster may, however, be biased by transit time. Including shorter transit times would reduce the ability to detect insects with low WBFs, whereas including longer transit times in order to resolve lower WBFs would disfavor perpendicular interceptions and sagittal observations, since detection ranges for species (and thus probe volumes) are likely to differ for transverse and sagittal observations. Moreover, clusters which have the roughly the same WBF can also differ in strength of even- and odd overtones (Fig. 4). This phenomenon is exemplified by cluster pairs such as C19 and C20 as well as C31and C32 (215 Hz and 250 Hz respectively) retrieved from the same day at the bush site. Such clusters could potentially arise from the same species and sex observed from different aspect angles. For both the C19–C20 pair and C31–C32 pair, the cluster with strong odd harmonics has a higher number of observations and has a longer detection range. Whereas cluster C31 and C32 have similar daily pattern likely representing one species, clusters C19 and C20 have distinct daily activity patterns, and could either represent distinct species or reflect different headings during the day. Generally, our understanding is that strong odd harmonics arise in observations from anterior/posterior aspects since the cross section is smaller in the transverse plane^26,37,39,51^. Therefore, the higher numbers and longer detection ranges of clusters with strong odd harmonics is counter intuitive. Potentially, this could arise if wing surface normals are more likely to produce specular reflections during upstroke than at their extreme angles.

**Fig. 5:**
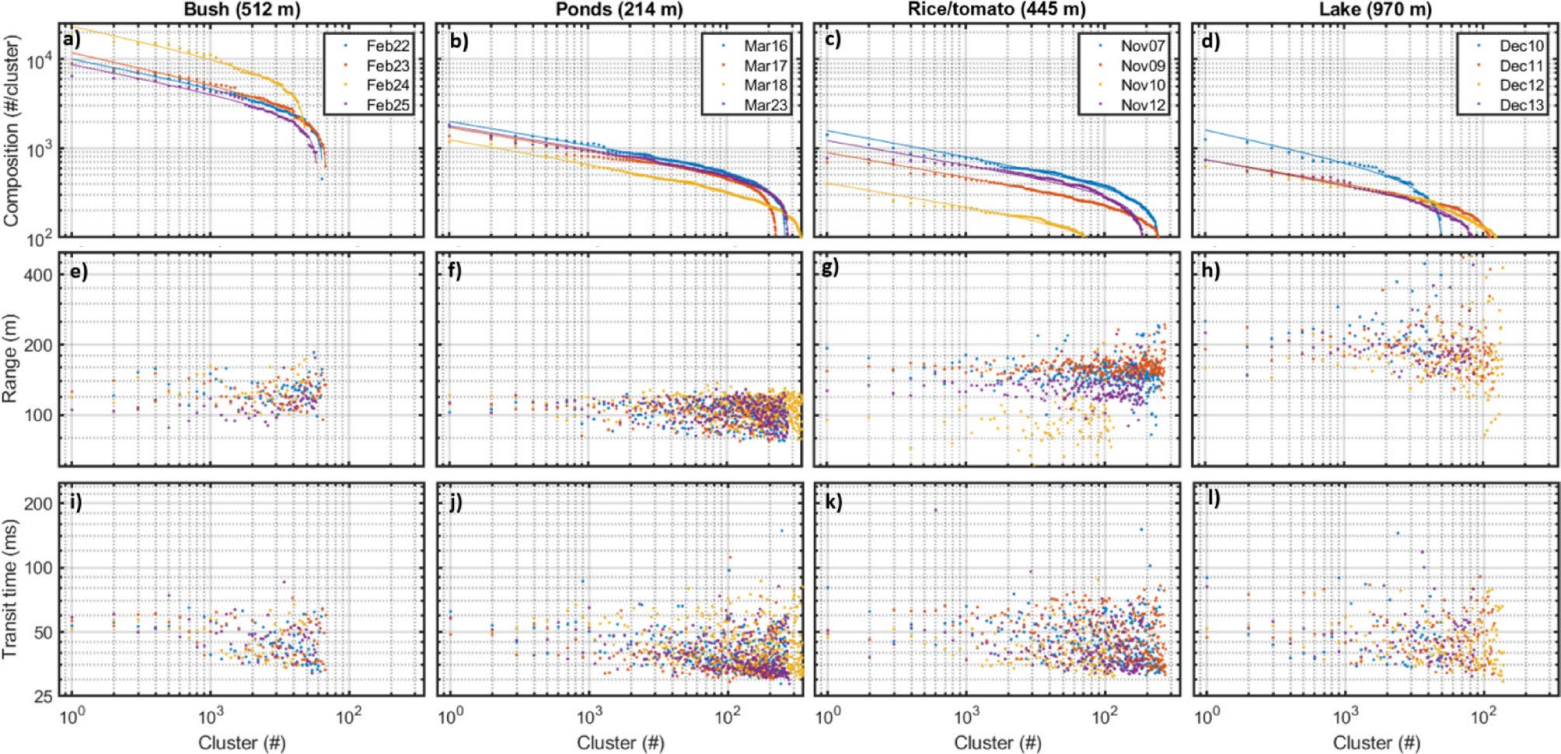
Cluster composition, detection range and transit time for each day and site. Top row: The number of insects per cluster as a function of cluster rank. The solid lines indicate approximation by model in Eq. 5. Mid row: the mean range from the lidar at which insects in a cluster are detected as function of cluster rank. Bottom row: The mean transit time for insects in each cluster for each cluster rank.

Ideally, the distribution of observation among clusters would be equivalent to the SAD, but more realistically the evenness of the distribution of cluster assignment co-varies with the SAD evenness. When clusters are sorted by descending observation number, we can accurately describe cluster abundance distributions by Eq. 4 (see “Methods” section) with explanation grades of ${\text{R}}_{{{\text{adj}}.}}^{2}$ > 99% for all sites and days (Fig. 5a–d, top row). Equation 4 is equivalent to SAD models, and the estimated composition slopes retrieved by fitting the equation are similar to those reported in other diversity studies^50,52^. We cannot establish any significant correlation between the number of observations assigned to a cluster and the average observation range for that cluster (Fig. 5e–h, Supplementary Table S1), implying that the higher number of observations in the most common clusters are not explained by a larger probe volume resulting from them being detected at larger distances. We could only see a weak indication that the average transit times of observations within a cluster is higher for common clusters (Figs. 5i–l, S1) according to Genoud et al.^24,53^.

**Fig. 6:**
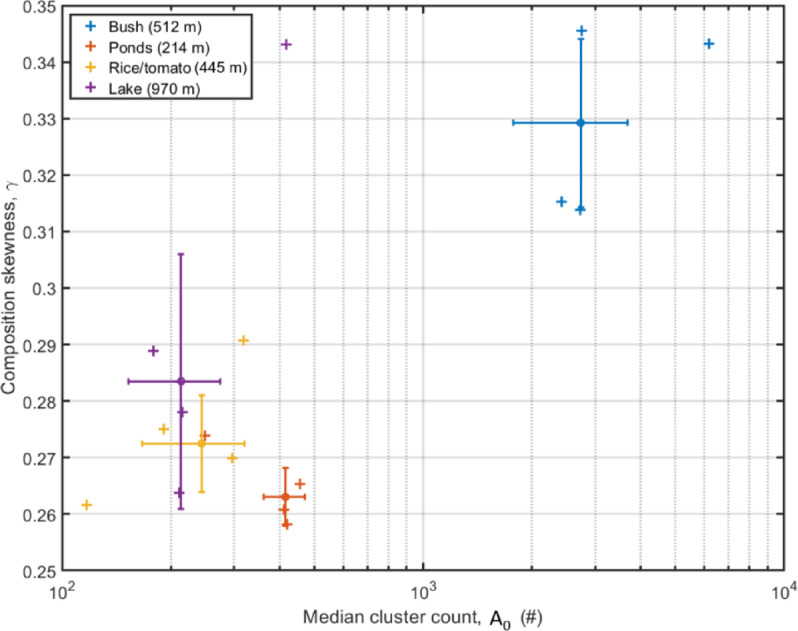
Compositional parameters, median cluster count and unevenness. The cluster assignment and composition from each day and site can be described by two parameters, the median cluster count, *A*_*1/2*_, roughly relating to the total count, and unevenness, *γ*. The two sites with the highest species richness, the pond and rice/tomato sites, display most even composition whereas the more species poor sites have a composition dominated by a few clusters. In particular, the bush site features fewer clusters than the other sites.

By parametrizing the cluster compositions from each day and site using Eq. 4 (“Methods” section, Supplementary Table S1), we recovered skewness. Within sites, the composition slopes are rather consistent among days (Fig. 6). The bush site had the highest number of observations but lowest diversity, and thus had a high skewness, *γ*, reflecting that it was dominated by few clusters (Fig. 6). In contrast, the pond site with the highest diversity had the most even distribution. Interestingly, the rice/tomato and lake sites displayed distinct patterns of diversity (Fig. 2) but their evenness could not be differentiated (Fig. 6). The diversity index *NoC* and the skewness *γ* can thus provide complementary information about the insect community.

Naïvely, one would like to think of insects entering an invisible beam over the fields as an unbiased assessment of the activity in the probed airspace. As we already eluded, some insect species are small, others large, some are dark and other bright. Some species have specular and resonant wings for the laser wavelengths and others have diffuse and omnidirectional scattering wings. To complicate the matter, flat specular^54,55^ scatter and omnidirectional^56^ backscattering is not attenuated equally over distance. When there are no specific preferred locations along the transect, the detection range distributions appear as in Figs. 1i–k and 4b, d, f, h. At close range, the numbers are reduced because of incomplete overlap between beam and field-of-view. This effect was described previously using the same Ivorian lidar^57^. When approaching the detection limit, *r*_*det.(c)*_, for a species, the number can be explained by Eq. 5 (“Methods” section). For the cases which were manually fitted (C19, C20, C30 and C31 on Feb23, see Fig. 4) the explanation grades were in the order of ${\text{R}}_{{{\text{adj}}.}}^{2}$≈90% (Fig. 4). The range attenuation was found to be spherical (*α ≈ 2*).

To test if detection range was accountable for clusters with small number of observations assigned to them, we plotted the clusters mean range in respect to their descending abundance rank (Fig. 5; middle row). We could not find any significant relation between average observation ranges of the cluster and the number of observations assigned to it. It would be reasonable to think that detection range for small species is short and detection range for larger species long, thus the probe volume would be larger for larger species, resulting in an overrepresentation of larger species. On the other hand, the biomass spectra dictate that the smallest species are the most numerous; these two effects may partly cancel out each other. Therefore, the listed bias effects from species specific probe volumes would reshuffle species in the sorted species abundance distributions, even in a fictive situation with perfect specificity.

Similar to range biases, the noise spectrum and thus detection limits are not the same during day and night as they are affected both by sunlight and atmospheric turbulence^58^. We expect sunlight and atmospheric turbulence from convection cells^58^ to slightly raise noise floor such that less small and far insect would be detected. Rain showers currently flood our detection algorithm, and we disable insect detection during rain although insect can fly in rain^59^. Wind would increase ground speed, shorten transit time and worsen frequency resolution. One interesting philosophical idea proposed by Genoud et al.^53^ is that aerial density should be deduced by weighting the observations by their transit times. We investigated cluster sizes in respect to the mean transit times and found weak correlations except for at the lake site (Fig. 5; bottom row, Supplementary Table S1). The lowest observable frequency and frequency resolution are both determined by transit time, and thus the width of the probe volume. Short observations generate the least specific signals and could be pooled together in unspecific clusters providing little information about diversity.

**Fig. 7:**
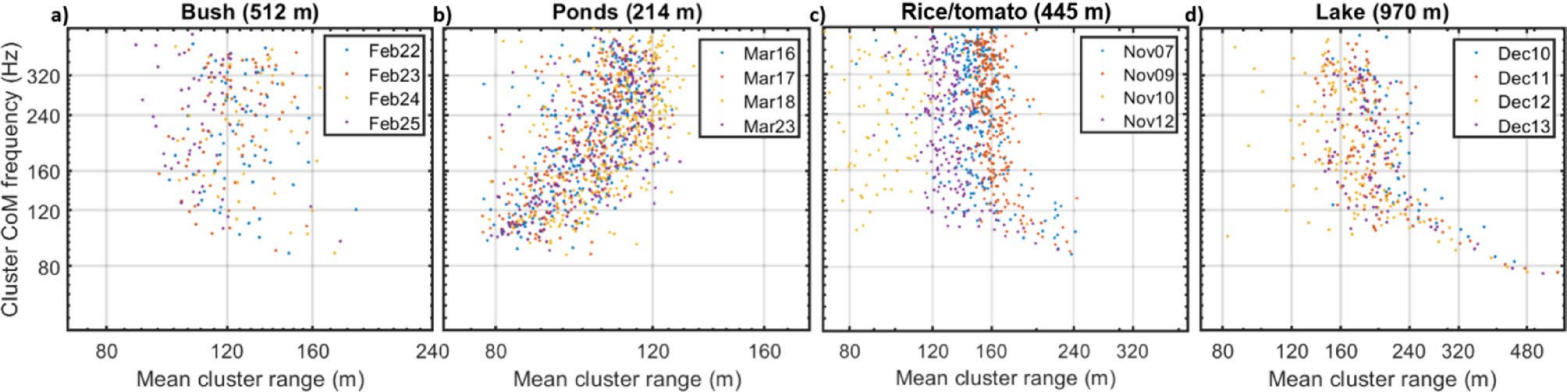
Dependence of detection range and thus probe volume in respect to frequency content. The relation is arbitrary, in the case of the bush-, rice/tomato- and lake- sites there was no difference in mean detection ranges in relation to mean modulation frequencies, *f*_*CoM*_, > 120 Hz, but clusters at the far ranges, *r* > 200 m, have comparatively much lower frequency (*f*_*CoM*_ < 120 Hz) and thus unspecific content. For the pond site higher frequency content was detected further away.

Finally, we investigated range biasing in respect to the frequency content of the clusters. The centroid power spectra were reduced to a single value by the first statistical moment. Accordingly, the center-of-mass frequency, *f*_*CoM*_, was obtained from each cluster for each day and site. These frequency values were correlated with the average observation ranges of the clusters (Fig. 7, Supplementary Table S1). We would like to emphasize that both range and frequency are double bound, thus their mean value could only assume value within range of the site and frequency range of the instrument and threshold for transit times.

The bush site showed no relation between *f*_*CoM*_ and mean cluster range, implying that distinct oscillation spectra can be assumed to have similar probe volumes. For the pond site, we encounter a significant positive correlation of 54% (Supplementary Table S1) between cluster range and higher frequency content (i.e. high WBF or strong harmonics). This aligns with the idea that specular reflections produce high frequency content^27,60^ and transmit over longer distances^54^, the same phenomenon which makes it possible to signal to an airplane by reflecting sunlight in a wrist watch. For the rice and tomato site, the transects are short and variability in cluster mean ranges are limited. An effect for *f*_*CoM*_ < 120 Hz gives rise to a significant negative correlation of − 22% (see Fig. 7c). This effect is more pronounced at lake site (Fig. 7d), where it gives rise to a negative correlation of − 48%. This implies that clusters observed over the lake are exclusively unspecific signals without clear WBFs and have modulation spectra governed by a rapid drop in power. It is possible that the lake habitat is dominated by larger insects and predators, whereas smaller insects with higher WBFs prefer shores. We have previously detected similar patterns^61,62^.

**Fig. 8:**
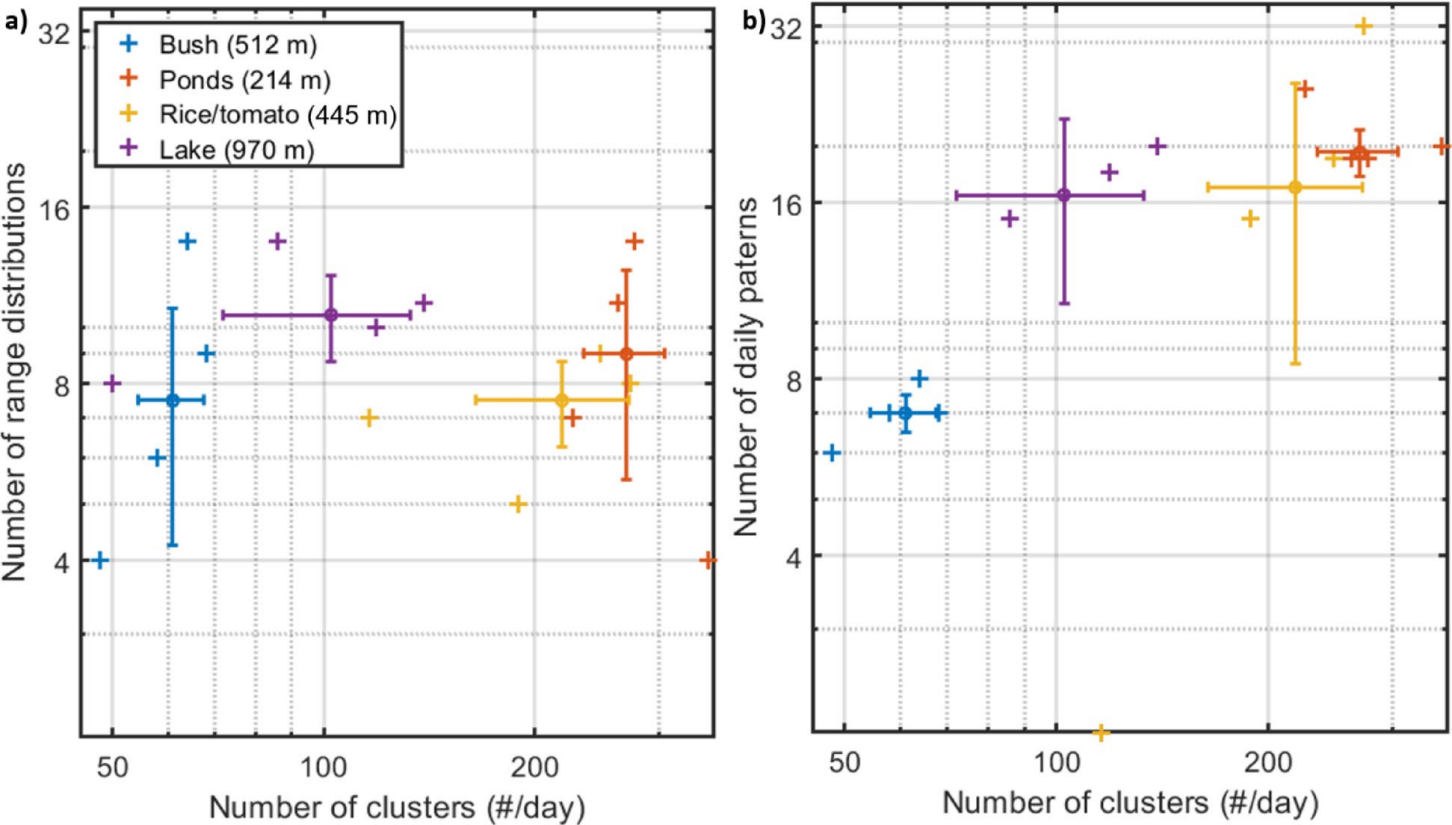
The number of unique range- and temporal distributions for the four sites. Hundreds of distinct oscillatory power spectra could be discerned from noise, but some clusters could represent multiple species and multiple clusters could arise from a single species. The number of distinct spatial (**a**)—and daily (**b**) patterns are much lower but confirms that multiple species with distinct sizes, landscape preferences and niches in terms of light levels, temperature and humidity during the day.

One complementary way to assess if inferred clusters represent distinct taxa is to investigate if clusters differing in modulation spectrum from the wing dynamics also differ in range distribution or daily activity pattern (Fig. 3, bottom row). Such differences would shed light on to which extent the recovered clusters prefer specific habitats, light levels or temperatures niches. To investigate cluster specific spatial- and temporal preferences ranges, histograms with 50 bins and 5% relative range resolution (matching the lidar accuracy) were calculated for all clusters for each day and site. Similarly, daily activity histograms were calculated using 96 time bins of 15 min width. We applied identical HCA cluster criterion as in Eqs. 1 to 3, but this time to the spatial- and temporal distributions rather than to the frequency content, illustrating the diversity of unique range and time distributions (Fig. 8).

The unique range and time patterns cannot exceed the half the number of detected clusters in Fig. 3 (the half factor arises from the median de-trending, Eqs. 2–3). In practice, the unique spatial and temporal distributions for the clusters are much lower than the cluster numbers (Fig. 8). Overall, the range distributions do not differ much between the sites. This could also be expected from the featureless total distributions (Fig. 2; middle row). Presumably, the identified unique range distributions predominantly relate to detection ranges for different clusters. The lake habitat, where the lake shore constitutes a distinct topographic feature, displays slightly more range unique distributions though, illustrating that it is possible to detect microhabitat preferences using this approach. At all sites, the beam was elevated over ground, resulting in low variation in topographic features. A beam trajectory closer to microhabitats such as plants, waterbodies or alternating between sun and shade, would most likely result in more unique range distributions.

The number of unique daily activity patterns are somewhat larger than the number of unique range distributions for three of the four sites, the bush site being the exception. Most sites display around 16 different daily activity patterns (Fig. 8b), which has wide-ranging implications for the design of sweep nets or rotation trap sampling efforts to detect biodiversity. Less than 16 daily sampling occasions could imply that species are missed, or that their relative frequency or numbers are misrepresented.

## Discussion

This comparative entomological lidar work is aimed at describing differences between sampling sites and assess the consistency across sampling days to assess the robustness of the estimates. We report an impressive total number of insect observations for a single study, counting 1.7 million observations during the 16-day duration, with up to 347 thousand daily observations and up to 353 differentiable clusters derived by classification of photonic signals for a single day and site. Lidar studies have previously recorded 312 thousand insects in 4 days^36^, and 12 classes have previously been distinguished by the Ivorian lidar^43^ also deployed in this study. A transmittance-based approach has previously been shown to discern 5 classes based on clustering of WBF of wild flying insects^40^, and approximately 30 species were retrieved in a study using another photonic sensor^44^. The number of observed insects in this study is high compared to studies using non-photonic approaches. For instance, 1.5 million insects were detected by radar during a 4 month period^63^, and up to 6 classes of insects have been distinguished by radar^64^. Malaise trapping has recovered 1 million insects during a 4-year period^21^, with consecutive barcoding and extrapolation estimating that 94 thousand insect species are documented. Thus, other state of the art methods have both advantages and limitations, and photonics classification can provide a powerful complement to these approaches.

We propose a simple, robust and parameter free criterion for estimating the number of unique photonically observed clusters from pair-wise hierarchical clustering. The current lidar instrument and algorithm has the potential to discern between 50 and 350 clusters (*NoC*s), depending on the local diversity at the sampling site. We found consistent and distinct differences between four study sites, as well as differences among the respective days when detection was performed. The differences between sampling days are likely be even stronger under sampling over a longer period with more pronounced weather differences.

Again, we emphasize that a single species can produce multiple signal types and thus *NoC*s since wing beats change with environmental conditions^40^, are distinct for the sexes^29^, change with payload such as eggs^42^, pollen, nectar^41^ and blood and that the aspect of observation change the relative strengths between their even and odd harmonics^26,28,38,39^. Furthermore, there will be a limit for the maximal number of distinguishable species with a certain instrument. This saturation level depends on both the signal–noise ratio, transit times and sample rate, the sophistication of the instrument in terms of wavelength-^65,66^ or polarization bands^61^, and the sophistication of the algorithm, for example implementation of waveform- or phase sensitive comparisons^67,68^. By inspection of the centroid modulation spectra we detected signals consistent with several clusters resulting from the same species and sex observed from different angles, whereas other clusters do not display clear WBF modulation and hence presumably pool multiple species. The number of clusters as described here should therefore be understood as a maximum number of signals that the lidar instrument and algorithm can discern from noise. This number is lower than the number of species present but still reflects relative species richness^37^. We also exploited the range distributions and daily activity patterns to set a minimum number of species that can be differentiated in this study.

Although we cannot expect a 1:1 relation between *NoC* and species richness, we can expect *NoC* to correlate with species richness, as demonstrated by Rydhmer et al.^44^. Although Rydhmer’s study was based on smaller distributed photonic sensors rather than a lidar, the algorithm is fairly similar to the one employed in is study. The numbers of clusters retrieved in this study are considerably higher than those reported by Rydhmer and colleagues based on studies in five Scandinavian habitats, where insect observations and catches were accumulated resulting in approximately 10^2^ catches and 10^3^ signals each week^44^. Further, Rydhmer et al. reported saturation level of approximately 40 known species in flight chambers. This means that photonically estimated richness likely will be underestimated in tropical forests, as species richness in tropical virgin forest can exceed 10^4^ species^10^.

Overall, the estimated *γ* values are low (too even) compared to surveys with Malaise traps and manual species identification^44,45,69^. Typical *γ* exponent values for Scandinavian Malaise studies is ~ 1.5 with minimal deviations for sites and seasons^44,45,52^, even if the dominant species are distinct between sites. The cluster unevenness and the true species unevenness are presumably related, but we cannot determine their relation, as some species may be pooled in dominant unspecific clusters or multiple clusters arise from the same species as discussed above. Imprecise methods would cause bias towards more even SADs (analogous to that photon counts from a low-resolution spectrometer yields spectral lines that are blurred together). Further comparisons of lidar data to ground truth data sets to validate the lidar based inferences, would be valuable for understanding the relationship between cluster- and species numbers. Obtaining species specific lidar signals from known specimens through controlled releases has proven inefficient due to the high number of observations needed to cover different observation aspects^26^. Larger amounts of lidar signals from known species can be retrieved in cage experiments where enclosures contain only one known species^28,29,44^. Indirect comparison to Malaise trap catches placed at the same sites as the lidar as in Rydhmer et al.^44^ provide a promising approach to validate the correspondence of lidar data to traditional approaches, making it more valuable for conservationists. Finally, improved light scattering models associating physical microstructure features of specimens to expected lidar signals as done in pilot studies^70,71^ provides a promising venue to improve the understanding of what species are observed in lidar data.

We found an interesting discrepancy between insect abundance and cluster diversity, where the site with largest number of insects was not the most diverse site in terms of number of distinguishable clusters, as would be expected from large scale studies on the relationship between insect abundance and diversity^49^. Instead, the site with the highest insect count had the lowest diversity of distinguishable clusters with a composition dominated by a few clusters. While species abundance distributions are reported to be stable to low-intensity disturbance^52^, abundance distributions differ among taxonomic groups^52^, and have been shown to be affected by land management differences^72^. The low number of clusters in spite of extremely high number of observations at the bush site also suggests that the observations retrieved using lidar can almost exhaustively sample a community, and derive relative abundance estimates for all distinguished clusters. It is important to acknowledge that there are likely cryptic species present within each cluster, and that the sexes may be split into distinct clusters. Regardless, there is good evidence that the number of oscillation-clusters is a good representation of the biodiversity in the area^44^. Furthermore, the digital signal processing pipeline and adaptive self-referencing cluster criterion (Eqs. 2–3) can circumvent a lot of the challenges associated with manual morpho-species designation^9^ (also known as taxon surrogacy^73^).

We investigated possible biases derived from detection range issues, transit times and frequency content. In one site, we found that higher frequency content was detected further away. For two of the sites we found that clusters with frequency content below 120 Hz were exclusively detected at far distances, whereas detection of clusters with wing beat frequencies higher that 120 Hz was unrelated to detection range (Fig. 8). For extended lidar transects, we can expect different detection ranges and thus probe volumes for large/small, dark/bright, glossy/matte species. Furthermore, species wing thickness resonates with the lidar wavelength^27,33,34^. The theory of specular lidar targets is fascinating^54,74^ but not yet well established for insect wings, as more work is needed to estimate species specific detection ranges and prove volume sizes. Jointly, these biases mean that species could be swapped in the ranked SAD (Fig. 5), depending on their specific detection ranges.

There are several venues for improving entomological lidar specificity. From experience, the number of discernible clusters increase by implementing polarization bands^61^, although the improvement is marginal. Incorporating multiple wavelengths bands^28,39,44^ providing additional information on melanization^65^ and wing membranes^33,34^ has an even higher potential to contribute to distinguishing even more clusters.

We emphasize that the power spectrum is not a complete description of the lidar retrieved waveforms in *I(t)* because phase information is lost. In addition, the power spectra can suffer from side lobes whenever the insect transit envelope through the probe volume deviates from Gaussian and frequency folding due the low *f*_*Nyq.*_. Improvement, without increasing instrument complexity, could include phase- or waveform sensitive pair wise comparison algorithms to better exploit retrieved information. We encourage the bioacoustics and signal processing communities to download the data and develop such approaches. An interesting future direction for the lidar would be to improve the understanding how specular reflections and frequency content is transmitted over distance, improving classification power.

In this study insect diversity was recorded over a period of several months, and sampling at the different study sites was not randomized. Thus, we cannot be certain that the differences encountered between sites do not partly reflect seasonal differences in insect composition. While the vegetation greenness at the sites did not differ between November and March (Figs. 1m–p, S1–S4), even tropical insect species may have distinct yearly phenologies, and different species may therefore be recorded during different times of the year^44^. Since our aim with this study is to demonstrate how entomological lidar can assess consistent differences in insect abundances and diversity estimates among sites, the implications of this design are small though. Having established this, our study clearly demonstrates the possibility to use lidar as cost-effective mean of non-invasive monitoring of insect communities. Moreover, the approach is feasible to apply even in regions with limited research budgets which are strongly underrepresented in insect diversity surveys^75^.

In conclusion, automated in situ assessment of biodiversity would have tremendous potential to evaluate and improve conservation planning. The work presented here shows that lidar has the potential to provide data that can be used to calculate richness as well as solid proxies for commonly used diversity indices such as Shannon–Wiener and Simpsons indices^76,77^. Entomological lidar thus provides a very promising biodiversity monitoring approach that can contribute unique data shedding light on insect abundance and diversity.

## Methods

### Lidar transects and sites

Four sites in vicinity of the Félix Houphouët-Boigny National Polytechnic Institute in Yamoussoukro, Ivory Coast were chosen for lidar observations. Yamoussoukro is below the arid Sahel region, but somewhat dryer than the costal region and harbors a range of habitats including bush, scrubs and small scale agricultural patches. Climatically, Yamoussoukro features a main rain season from March to July and a smaller rain season in September and October. Here, the same lidar system was deployed at multiple sites. The sites were deliberately placed in different habitat types (Figs. 1, S1–S7) that can be expected to display distinct insect abundance and species richness. Insects were recorded for four consecutive days at each site during the dry season from November 2021 to March 2022. As the sites were monitored at different time points, we cannot with certainty disentangle the difference between sites and progression into the dry season. However, this was of minor importance, because our aim was to compare days to each other, regardless of whether these differences were due to sites or seasonality. Additionally, the dry season is climatically stable, and the differences between the sites are pronounced. The rice/tomato site in November was not greener than the pond site in March (see Figs. S1–S4). Therefore, we refer to the four lidar transects by their site identity rather than their recording date (Fig. 1). One day was windy (17th March) and one day was rainy (10th November), otherwise the weather was stable, and recordings could be carried out continuously for 24 h.

### Entomological lidar instrument

The Ivorian entomological lidar system was previously described in other studies^43,57,71,78,79^; see also Fig. S5. The lidar also resemble other system^27,41,65,80–82^. Briefly, an invisible near infrared beam from a 3W, 808 nm laser diode was transmitted over an area and terminated on a board with black neoprene (Fig. S6). The beam expander diameter was Ø102 mm, the near limit of the system was 40 m, and the beam was typically terminated after several hundred meters. The backscattered light from insects going through the beam was collected with a Newtonian telescope equipped with a spectral band pass filter and a tilted linear CMOS detector in the focal plane (according to Scheimpflug condition and the hinge rule). In this study, the detector was set to acquire 3500 full waveform echoes/s, and the laser diode was modulated in synchronization with the detector to enable real time optical background subtraction and operation throughout the day. This implies that the effective sample rate, *f*_*s*_, of the system is 1.75 kHz and thus a Nyquist frequency, *f*_*Nyq.*_, of 875 Hz.

### Entomological lidar data sets

The lidar was set to acquire raw data files in the form of 16 bit backscattered intensities by 2048 pixel (range bins) and 35,000 exposures (17,500 shots). Each file represents 10 s, has a size of 143 Mb, and is stored on a USB terabyte disk. Every day in this study corresponds to some ~ 8000 datafiles and ~ 1.5 Terabyte of data, making the total raw data analyzed in this study amount to ca. 24 Terabyte. Insect observations were cropped out of the raw data as described in previous work^36,82,83^. Briefly, individual observations are defined as connected islands in a Boolean time-range map where backscatter exceed its own median plus two interquartile ranges (IQRs) for the given range. This cropping reduces the data approximately by a factor 1:1000, depending on insect abundance. Many features can be extracted from such insect observations, *n*, but in this study we limited the parameters for each insect observation to; a range, *r(n)*, (in meters), a time stamp, *t*_*0*_*(n)*, (HH:MM) and a time dependent oscillatory backscatter intensity vector, *I(t,n),* in 16 bit. In addition, every time an insect observation was cropped from raw data, we also cropped an empty piece of real instrument noise, *I*_*ξ*_*(t,n)*, using an identical mask in same file and range but with distinct *t*. This is used as a negative control and to avoid biases because instrument noise may depend on mask size in terms of duration (exposures) and aperture (pixels), temperature, background sunlight levels, turbulence and thus range. The detected number of insects per minute over the course of the day varied from 1 to 1000 (Fig. 2a–d). The most likely transit time, *Δt*, was 25 ms (Fig. 2e–h). March 17th displayed a distinct slope because of wind (Fig. 2f) and November 10th had a reduced count because of multiple rain shower interruptions (Fig. 2g). The 25 ms transit time corresponds to 43 time samples, which allowed us to estimate the modulation power spectra, *P(f,n)* in 40 frequency bins from 1/25 ms = 40 Hz to *f*_*Nyq.*_ = 875 Hz, with bin spacing of 20 Hz and spectral resolution of *Δf* = 40 Hz. The power spectra were estimated by the Welch method using a Gaussian window of 40 samples lengths and a FWHM of 20 samples. Equivalently, we also estimated the power spectra of the noise, *P*_*ξ*_*(f,n)*, corresponding to the conditions for each observation. In total for all days and all sites 1,716,362 observations (~ 75% of all observations) exceeded a transit time of 25 ms.

### Hierarchical clustering of observations

HCA was previously applied to modulation spectra from entomological lidar^35,36,43^. In the current study, the dates and sites were evaluated independently and the pair-wise similarity distance, *D(a,b)*, between all the power spectra retrieved from the focal site and day was computed accordingly;

1 $$D\left( {a,b} \right) = \sqrt[2]{{\mathop \sum \limits_{{f = 40\;{\text{Hz}}}}^{{875\;{\text{Hz}}}} \left( {\log \left( {\frac{{P\left( {f,a} \right)}}{{\mathop \sum \nolimits_{{40\;{\text{Hz}}}}^{{875\;{\text{Hz}}}} P\left( {f,a} \right)}}} \right) - \log \left( {\frac{{P\left( {f,b} \right)}}{{\mathop \sum \nolimits_{{40\;{\text{Hz}}}}^{{875\;{\text{Hz}}}} P\left( {f,b} \right)}}} \right)} \right)^{2} }}$$

here *a* and *b * ∈ 1… *N*, *a ≠ b*, and denote two observation indices. The power spectra were auto-normalized such that only their different shapes are considered and not their absolute magnitude (which can vary a lot with range and position in the beam). In addition, the modulation power was logarithmized prior to calculation of the Euclidean statistical distances. This implies a fuzzy logical *and* operation across the spectral bins, and that all harmonic content of two observations must match to produce a similar pairwise distance. The pairwise distances were organized into a linkage tree *Z*_*(p)*_ for each branch,* p*. Which is a compressed form of *D*_*(a,b)*_ (since* p* ∈ 1…*N*−1 compared to the size of *D* which is *N²*). The branch linkages were sorted in descending order, thus starting with dividing the most different types of modulation spectra. In practice, *D(a,b)* is too large to compute (~ 1TB for site with highest abundance) and linkage, *Z*_*(p)*_, was instead computed in Matlab (Statistics and machine learning toolbox, MathWorks, USA), using the *‘ward’* and *‘save memory’* algorithm flags. Equivalent linkages were computed between the instrument noise fragments, *Z*_*ξ(p)*_, as a negative control and to avoid any obscure biases arising from cropping, instrument- or environmental noises sources. Both the linkage for the insect observations, *Z*_*(p)*_, and for the noise fragments, *Z*_*ξ(p)*_, displayed steady declines. To remove this trend, we compensated the linkages by a power law with an exponent, *β*, equal to the median slope of the linkages (sorted in descending order), see Eq. 2.

2 $$\begin{array}{*{20}c} {Z_{comp.\left( p \right)} = \left( {\frac{N - 1}{p}} \right)^{\beta } Z_{\left( p \right)} } & {, \beta = \left| {\frac{{{\Delta }\log Z_{\left( p \right)} }}{{{\Delta }\log p}}} \right|_{median} } & {, p \in \left\{ {1 \ldots N - 1} \right\}} \\ \end{array}$$

The median slopes, *β*, were slightly below ½ (square root) for all sites and days. Following this compensation, the linkages are comparable (Fig. 1m–p). Whereas the observational data display a decline, the noise control display flat plateau. A rapid drop is observed for the linkages when *p* → *N*. This effect is not related to biodiversity, but results from the dimensionality of the parameter space (40 DoF, the number of frequency bins in our case) and numerical precision of measurements and computation.

We identified the number of clusters for each day and site, *NoC*, as the number of compensated linkages exceeding the median plus one IQR of the same compensated linkages, see Eq. 3.

3 $$NoC = \mathop \sum \limits_{p}^{N - 1} \left[ {Z_{comp.\left( p \right)} > \left( {\left| {Z_{comp.\left( p \right)} } \right|_{median} + \left| {Z_{comp.\left( p \right)} } \right|_{IQR} } \right)} \right]$$

Similar procedures and criteria can be encountered in other recent entomological lidar studies^44,61,71^.

### Abundance distributions of clusters

Numerous analytical expressions have been developed for SADs^84^. To estimate a proxy for SAD, we sorted the number of observations, *A(c)*, for each cluster, *c* ∈ 1…*NoC*, for each day and site in descending abundance. The distributions was described by fitting this equation:

4 $$\hat{A}_{\left( c \right)} = A_{0} \left( {1 - \frac{NoC + 1}{c}} \right)^{\gamma }$$

where *Â*_*(c)*_ is the estimated counts assigned to each cluster, *A*_*0*_ is a scalar for cluster count and *γ* unevenness of cluster assignments. The coefficients *A*_*0*_ and *γ* are estimated by regression with confidence intervals of 2% for all sites and days. This model yielded explanation grades of ${\text{R}}_{{{\text{adj}}.}}^{2}$ > 99% for the compositions at all sites (Fig. 5). An unevenness of *γ* = *0* imply that all clusters are equally represented and a high *γ* value imply that composition is dominated by a few clusters and thus species.

### Detection range of clusters

When there are no topographical preferences along the transect, the range distribution of observation pertaining to cluster, *c*, can be approximated by:

5 $$\hat{A}_{{\left( {r,c} \right)}} = A_{\left( c \right)} \sqrt[\alpha ]{{1 - \left( {\frac{r}{{r_{det.\left( c \right)} }}} \right)^{\alpha } }}$$

where *A*_*(c)*_ is the abundance of a cluster within detection range, *r*_*det.(c)*_, and *α* describe the slope of the signal attenuation over distance which could differ for diffuse and specular species.

## Supplementary Information

Below is the link to the electronic supplementary material.

Supplementary Material 1

## Data Availability

The data is available as supplementary information for ecological interpretation and improvement of algorithms. https://lu.app.box.com/s/u2ffzcbukzle01ghv5w4dvxq4bp10fr5.
