# Supplementary material for: Lidar reveals distinct insect daily activity and diversity between habitats
Source: Sci Rep. 2025 Nov 21;15:43544. doi: 10.1038/s41598-025-27432-9 (PMC12695965; doi:10.1038/s41598-025-27432-9)
Supplement: Supplementary file 1 — Supplementary Material 1 [file 41598_2025_27432_MOESM1_ESM.pdf]

Supporting information

All data will be deposited at a data repository upon acceptance of the manuscript.

Fig. S1 – Bush site *(Drone images captured by authors)*

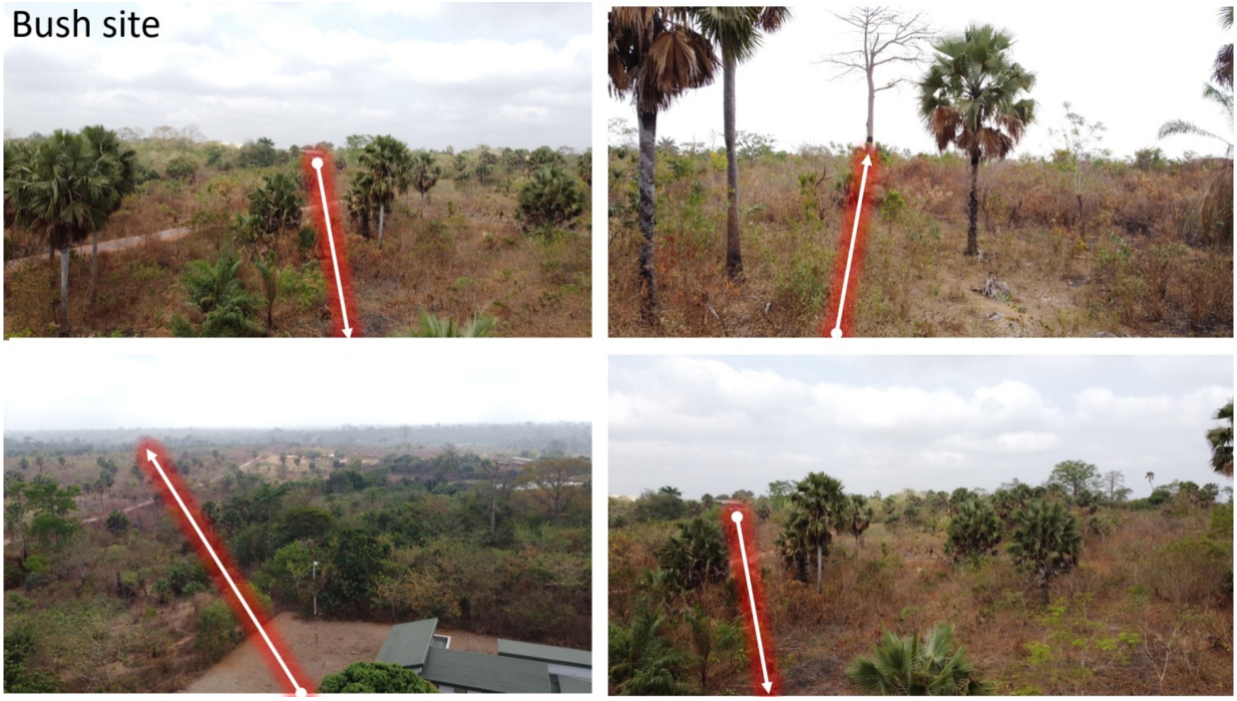

Fig . S2 – Pond site *(Drone images captured by authors)*

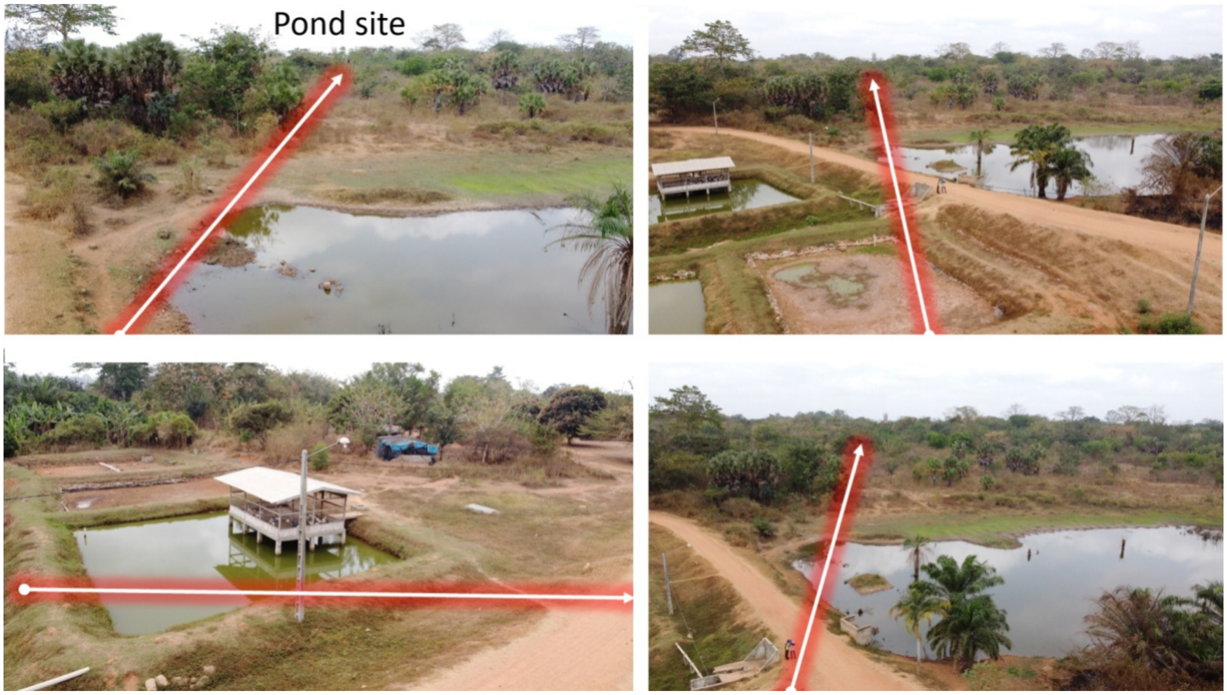

Fig . S3 – Rice/tomato site *(Drone images captured by authors)*

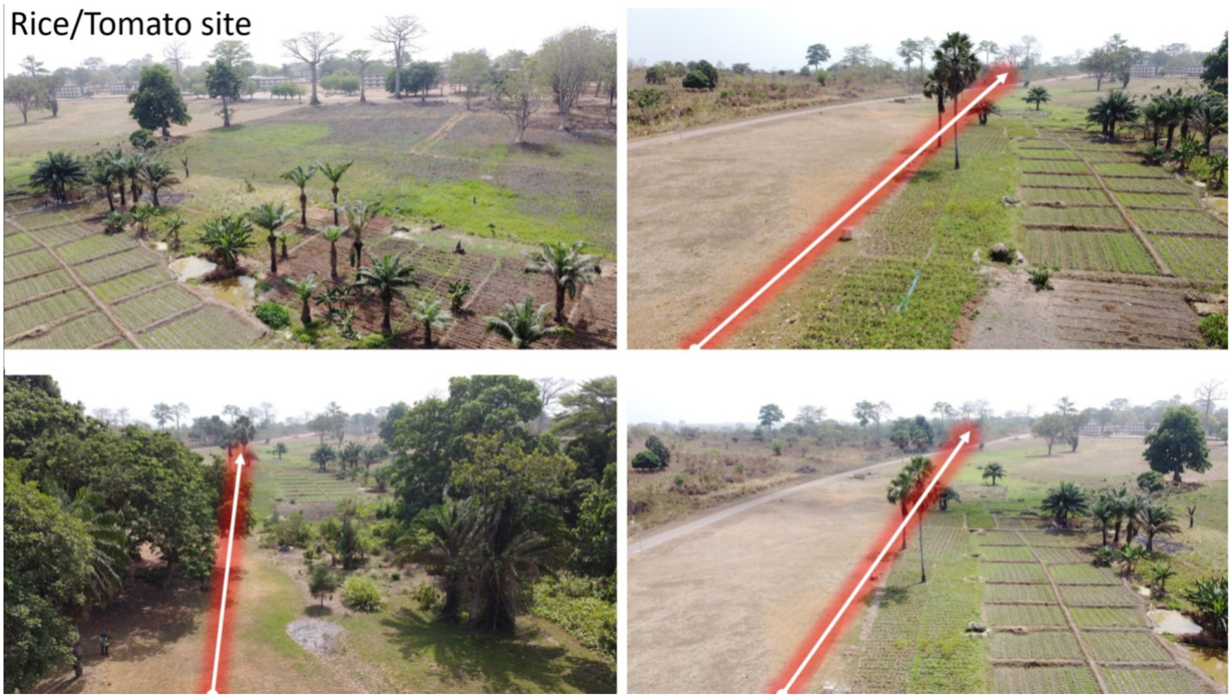

Fig. S4 – Lake site *(Drone images captured by authors)*

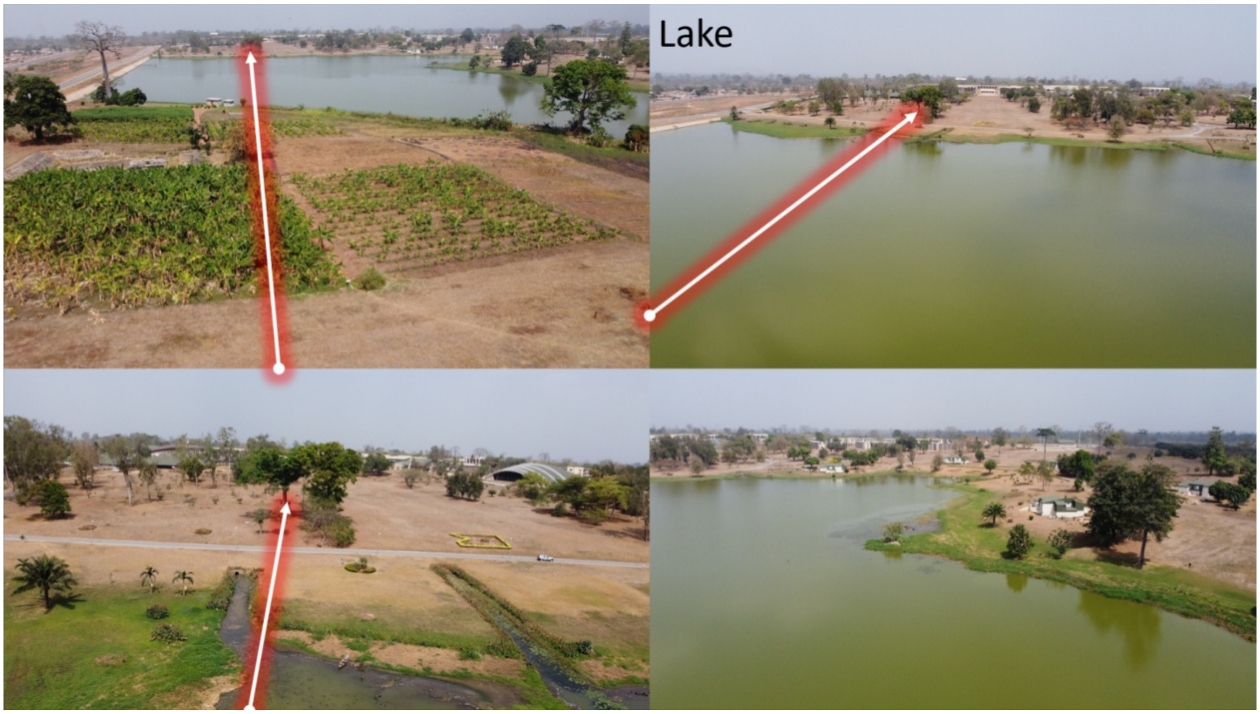

Fig . S5 – Ivorian entomological lidar

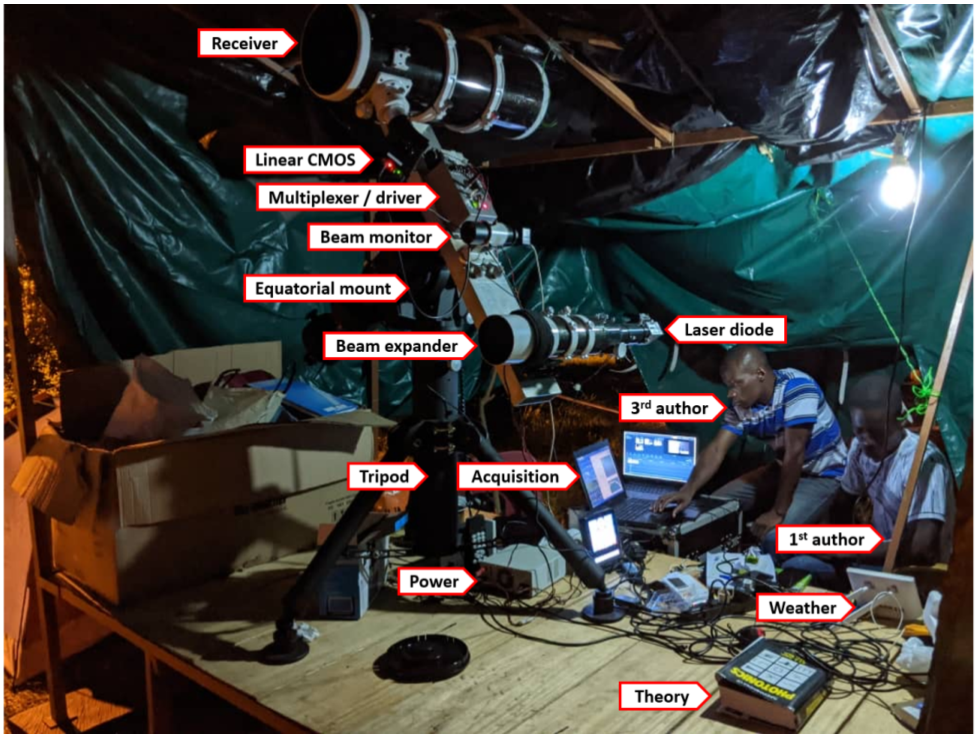

Ivorian entomological lidar, 2021

The measurements were not performed on human participants. The individuals appearing in Supplementary Fig. S5 are the first and third authors of the manuscript. Both authors contributed to the writing of the article and have given their consent for the use of their images in the manuscript.

Fig. S6 – Beam termination

Example of beam termination (lake site)

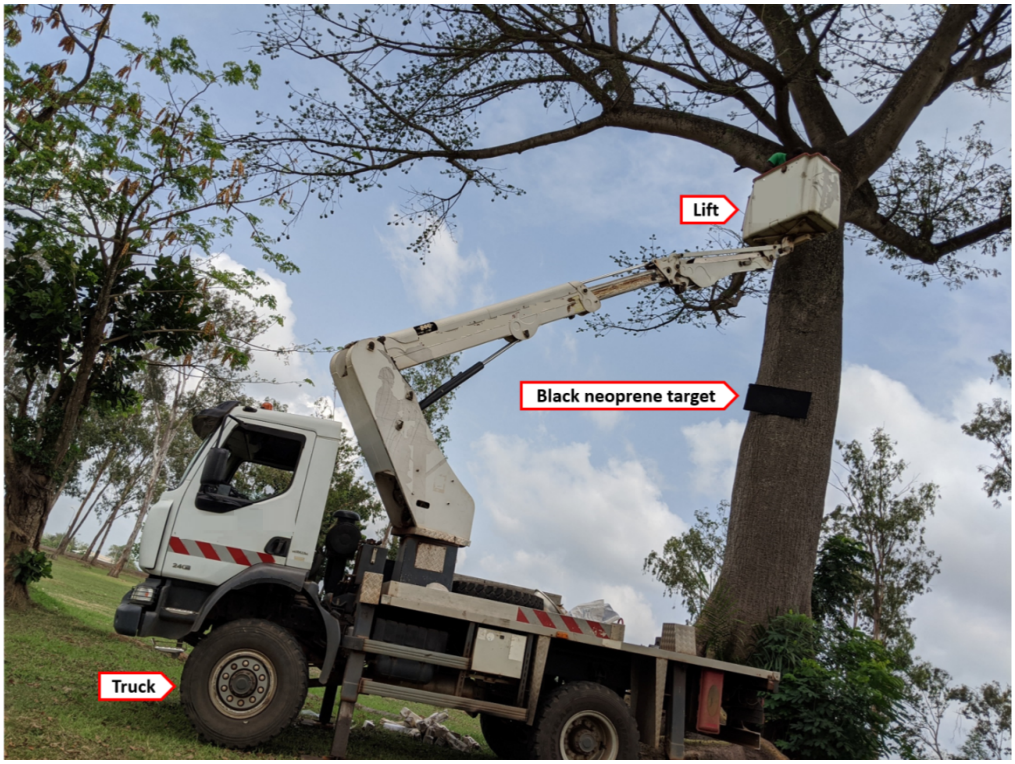

Fig. S7 - Site locations *(Map data: © Google, Maxar Technologies)*

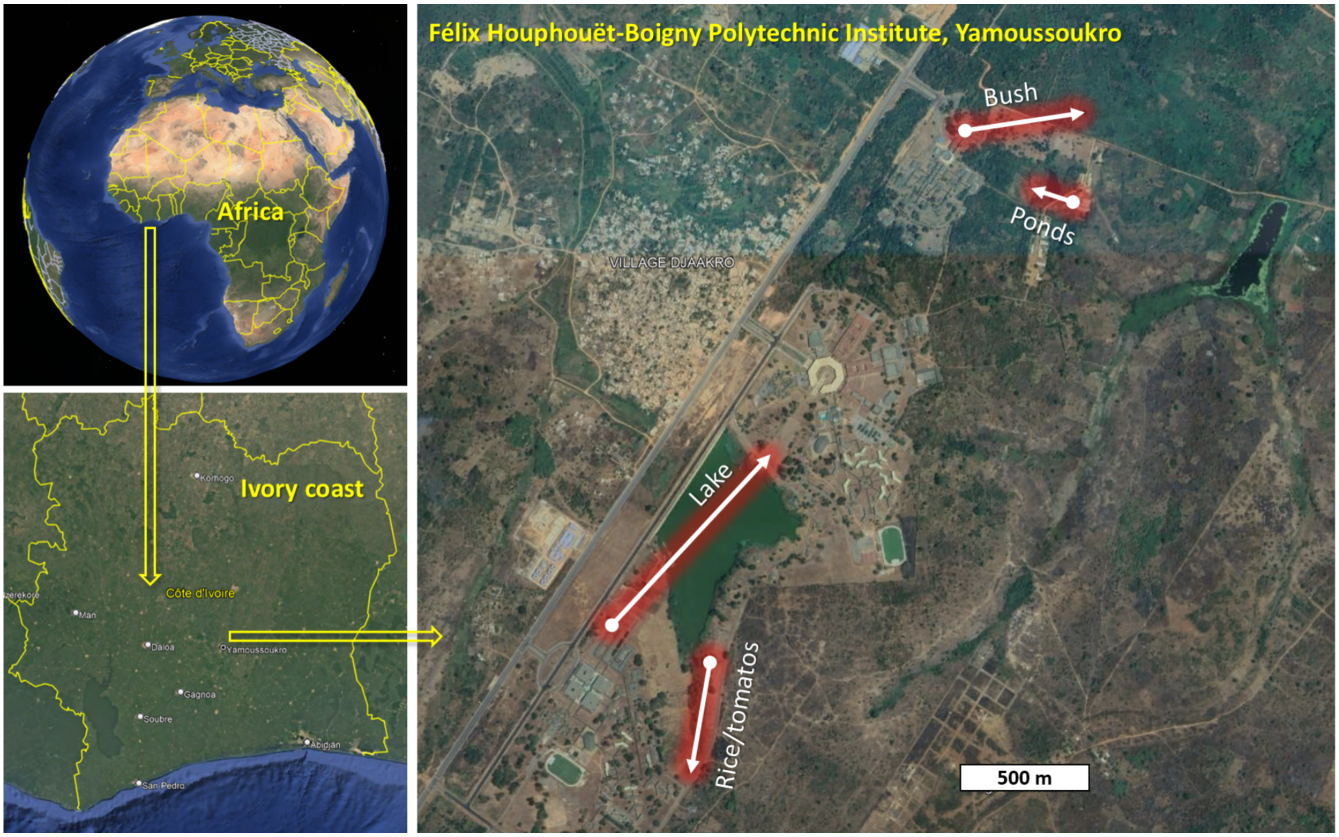

Site	Lidar			Termination			Beam
	Latitude	Longitude	Height	Latitude	Longitude	Height	Range
**Bush**	6°53’26.47”N	5°13’28.50”W	5 m	6°53’28.49” N	5°13’11.62”W	7 m	514 m
**Ponds**	6°53’17.07”N	5°13’21.02”W	3 m	6°53’16.25” N	5°13’14.01”W	8 m	214 m
**Rice/tomato**	6°52’13.92”N	5°14’03.56”W	3 m	6°51’59.66” N	5°14’05.55”W	7 m	445 m
**Lake**	6°52’19.05”N	5°14’16.66”W	3 m	6°52’43.24” N	5°13’56.28”W	9 m	970 m

SUPPLEMENTARY TABLE S1 - Observational summary

Site	Date	N_{obs}	β Eq.2	*NoC* Eq.3	γ Eq.4	A_0 Eq.4	Number of range distributions	Number of daily patterns	Correlation $N(c)$ vs. $\Delta t_{mean(c)}$	Correlation $N(c)$ vs. $r_{CoM(c)}$	Correlation $r_{CoM(c)}$ vs. $f_{CoM(c)}$
Bush	22nd Feb. 2022	198448	-0.49	64	0.31	2715	14	8	+37%($\rho<10^{-2}$)	+1%($\rho<10^{0}$)	-5%($\rho<10^{0}$)
Bush	23rd Feb. 2022	219572	-0.49	68	0.35	2747	9	7	+31%($\rho<10^{-2}$)	+6%($\rho<10^{0}$)	+25%($\rho<10^{-1}$)
Bush	24th Feb. 2022	346581	-0.49	48	0.34	6200	4	6	+19%($\rho<10^{0}$)	-13%($\rho<10^{0}$)	-14%($\rho<10^{0}$)
Bush	25th Feb. 2022	160927	-0.49	58	0.31	2424	6	7	+37%($\rho<10^{-2}$)	-41%($\rho<10^{-2}$)	-17%($\rho<10^{0}$)
Pond	16th Mar. 2022	133979	-0.48	263	0.27	456	11	19	+26%($\rho<10^{-4}$)	+20%($\rho<10^{-2}$)	+57%($\rho<10^{-23}$)
Pond	17th Mar. 2022	104738	-0.48	226	0.26	420	7	25	+10%($\rho<10^{0}$)	+21%($\rho<10^{-2}$)	+57%($\rho<10^{-20}$)
Pond	18th Mar. 2022	99143	-0.49	353	0.27	249	4	20	+28%($\rho<10^{-7}$)	+16%($\rho<10^{-2}$)	+50%($\rho<10^{-23}$)
Pond	23th Mar. 2022	126469	-0.49	277	0.26	411	14	19	+24%($\rho<10^{-4}$)	+26%($\rho<10^{-4}$)	+50%($\rho<10^{-18}$)
Rice/tomato	7th Nov. 2021	89573	-0.48	248	0.29	318	9	19	+22%($\rho<10^{-3}$)	+12%($\rho<10^{-1}$)	-39%($\rho<10^{-9}$)
Rice/tomato	9th Nov. 2021	58499	-0.49	273	0.28	191	8	32	+32%($\rho<10^{-7}$)	-13%($\rho<10^{-1}$)	-46%($\rho<10^{-16}$)
Rice/tomato	10th Nov. 2021	14943	-0.47	116	0.26	117	7	2	+15%($\rho<10^{0}$)	+9%($\rho<10^{0}$)	+38%($\rho<10^{-4}$)
Rice/tomato	12th Nov. 2021	62410	-0.49	189	0.27	296	5	15	+21%($\rho<10^{-2}$)	+4%($\rho<10^{0}$)	-46%($\rho<10^{-10}$)
Lake	10th Dec. 2021	24242	-0.50	50	0.34	418	8	0	+14%($\rho<10^{0}$)	-7%($\rho<10^{0}$)	-63%($\rho<10^{-6}$)
Lake	11th Dec. 2021	27869	-0.49	119	0.26	212	10	18	+13%($\rho<10^{0}$)	+5%($\rho<10^{0}$)	-46%($\rho<10^{-7}$)
Lake	12th Dec. 2021	28145	-0.48	139	0.29	179	11	20	+12%($\rho<10^{0}$)	-2%($\rho<10^{0}$)	-50%($\rho<10^{-9}$)
Lake	13th Dec. 2021	20824	-0.49	86	0.28	216	14	15	+13%($\rho<10^{0}$)	-2%($\rho<10^{0}$)	-41%($\rho<10^{-4}$)
